# A randomized controlled trial of virtual reality–based relaxation to reduce pre-exam stress among nursing students

**DOI:** 10.3389/fpsyt.2026.1859945

**Published:** 2026-07-10

**Authors:** Mohammed Owayrif Alanazi

**Affiliations:** Department of Nursing, College of Applied Medical Sciences, University of Bisha, Bisha, Saudi Arabia

**Keywords:** virtual reality, nursing education, test anxiety, stress management, digital therapeutics

## Abstract

**Background:**

Test-related stress and anxiety are common among nursing students and may negatively affect academic performance and well-being. Virtual reality (VR)–based relaxation interventions have emerged as a potential strategy to reduce stress; however, evidence regarding their feasibility and acceptability in pre-examination settings remains limited. This study aimed to examine the effects of a brief VR relaxation intervention on perceived stress and test anxiety among nursing students, and to evaluate the intervention’s acceptability and feasibility.

**Methods:**

This randomized controlled trial (RCT) evaluated the impact of a 10-minute, self-selected immersive VR relaxation session on nursing students’ perceived stress and test anxiety prior to a final practical exam. Participants (N = 52) were randomized into a VR intervention group or a control group. The intervention group chose from various naturalistic VR environments (e.g., beach, forest).

**Results:**

The sample (n = 52) was 55.8% female and 44.2% male, with the majority aged 20–21 years (63.5%). Most participants were in their third academic year (57.7%). The mean cumulative GPA was 3.60 ± 0.66. There were no statistically significant differences between the intervention and control groups in any baseline characteristics (all *p* >.05). The intervention group showed a significant reduction in perceived stress (*p* = .014) and test anxiety (*p* = .002). In contrast, the control group had no significant change in stress and anxiety.

**Conclusion:**

A brief, 10-minute immersive VR session shows promise as a supportive intervention for managing pre-exam stress and anxiety in nursing students. The findings suggest that integrating VR technology into nursing curricula offers a scalable solution to improve student well-being and cognitive readiness during high-pressure academic periods.

**Clinical Trial Registration:**

https://clinicaltrials.gov/study/NCT07574203, identifier NCT07574203.

## Highlights

- Brief immersive VR reduced pre-exam stress in nursing students.
- VR intervention lowered test anxiety compared to control group.
- Students reported high acceptability and ease of using VR.
- Intervention was feasible for short pre-exam implementation.
- Study demonstrates VR as a practical tool for nursing education.

## Background

Nursing students frequently experience significant stress before high-stakes assessments, particularly during clinical competency evaluations such as final practical exams. This stress has been shown to negatively impact learning outcomes, psychomotor performance, and emotional well-being (1, 2). Furthermore, elevated stress levels can impair focus, critical thinking, and clinical decision-making, especially in high-pressure environments like simulation or examination settings (3). In recent years, immersive Virtual Reality (VR) technologies have gained recognition as promising tools to support emotional regulation and stress reduction in both healthcare education and clinical practice (4, 5). VR provides an interactive, multi-sensory environment that can simulate calming natural scenes, tourist destinations, or other tranquil environments. Evidence shows that using short VR-based relaxation intervention can significantly reduce self-reported stress, anxiety, and even physiological indicators such as heart rate and blood pressure (6–8).

In nursing education, relaxation-based interventions have been shown to play a critical role in mitigating stress and enhancing students’ psychological readiness, particularly in high-pressure contexts such as examinations (4–6). Randomized controlled trials (RCTs) indicate that relaxation and comfort strategies can reduce pre-procedural stress, improve adaptation to demanding environments, and enhance self-confidence among nursing students (9). For example, an RCT involving 77 nursing students reported that those who received an instrumental music–based relaxation intervention experienced significantly lower stress levels and greater self-confidence compared with controls (9). Similarly, a study conducted prior to clinical examinations demonstrated that a brief, 10-minute immersive virtual reality (VR) experience significantly reduced both state anxiety and physiological indicators of stress among nursing students (n=22) (6).

The immersive nature of VR, especially when combined with nature sounds and ambient visuals, appears to stimulate relaxation through sensory distraction and emotional engagement (10). Moreover, emerging evidence indicates that allowing participants to select their preferred VR environment (e.g., beach, forest, or space) enhances engagement, perceived immersion, and sense of control, thereby enhancing the stress-reducing and relaxation effects of VR-based interventions (7). This aligns with findings that user choice within VR environments correlates with improved emotional outcomes and a stronger sense of presence (11). In nursing students, VR-based intervention has also been found to improve sleep quality and anxiety, supporting broader recovery and well-being (6).

Given this body of evidence, the present study was conducted to evaluate the effectiveness of a brief VR–based relaxation intervention delivered immediately prior to the final practical examination in a Critical Care Nursing course. The study aimed to assess whether a self-selected 10-minute immersive VR relaxation session could reduce perceived stress among undergraduate nursing students in Saudi Arabia. The intervention applied a learner-centered and personalized approach by allowing participants to select from a range of immersive VR environments, including tropical islands, beaches, deep ocean settings, African savanna landscapes, well-known international destinations (e.g., London, New York, and the Seychelles), and outer space, with the intention of enhancing engagement, immersion, and emotional regulation and thereby optimizing the stress-reducing effects of the VR intervention.

## Methods

### Design

This RCT was conducted as an exploratory study, with primary focus on within-group pre–post changes and the acceptability and feasibility of the VR intervention. Participants were randomly assigned to either a 10-minute immersive VR relaxation session (intervention group) or a quiet, non-stimulating room condition (control group).This study was reported in accordance with the Consolidated Standards of Reporting Trials (CONSORT) 2010 guidelines.

### Ethical considerations

Prior to conducting the study, ethical approval was obtained from the University Institutional Review Board (UB-RELOC H-06-BH-087). All participants provided informed consent before participating in the study. Participants were clearly informed that participation was entirely voluntary and that they could withdraw at any time without penalty. It was explicitly stated in the consent that participation or non-participation would have no effect on course grades, academic standing, or examination outcomes. The VR intervention was not part of the course requirements or any assessed academic activity. Recruitment, data collection, and consent procedures were conducted in a manner designed to minimize any perceived coercion and to ensure that students were able to make an independent and informed decision regarding participation.

### Sample

Participants were nursing students enrolled in the Bachelor of Nursing program at the University of Bisha and recruited between July 14 and 17, 2025. Eligible participants were male and female students currently enrolled in the Critical Care Nursing course, scheduled to undertake their final practical examination within 24 hours, and willing to provide informed consent. Exclusion criteria included a self-reported diagnosis of motion sickness, epilepsy, or any sensory disorder; prior use of VR for relaxation purposes; and the use of anxiolytic medications within the 24 hours preceding participation.

**Figure 1:**
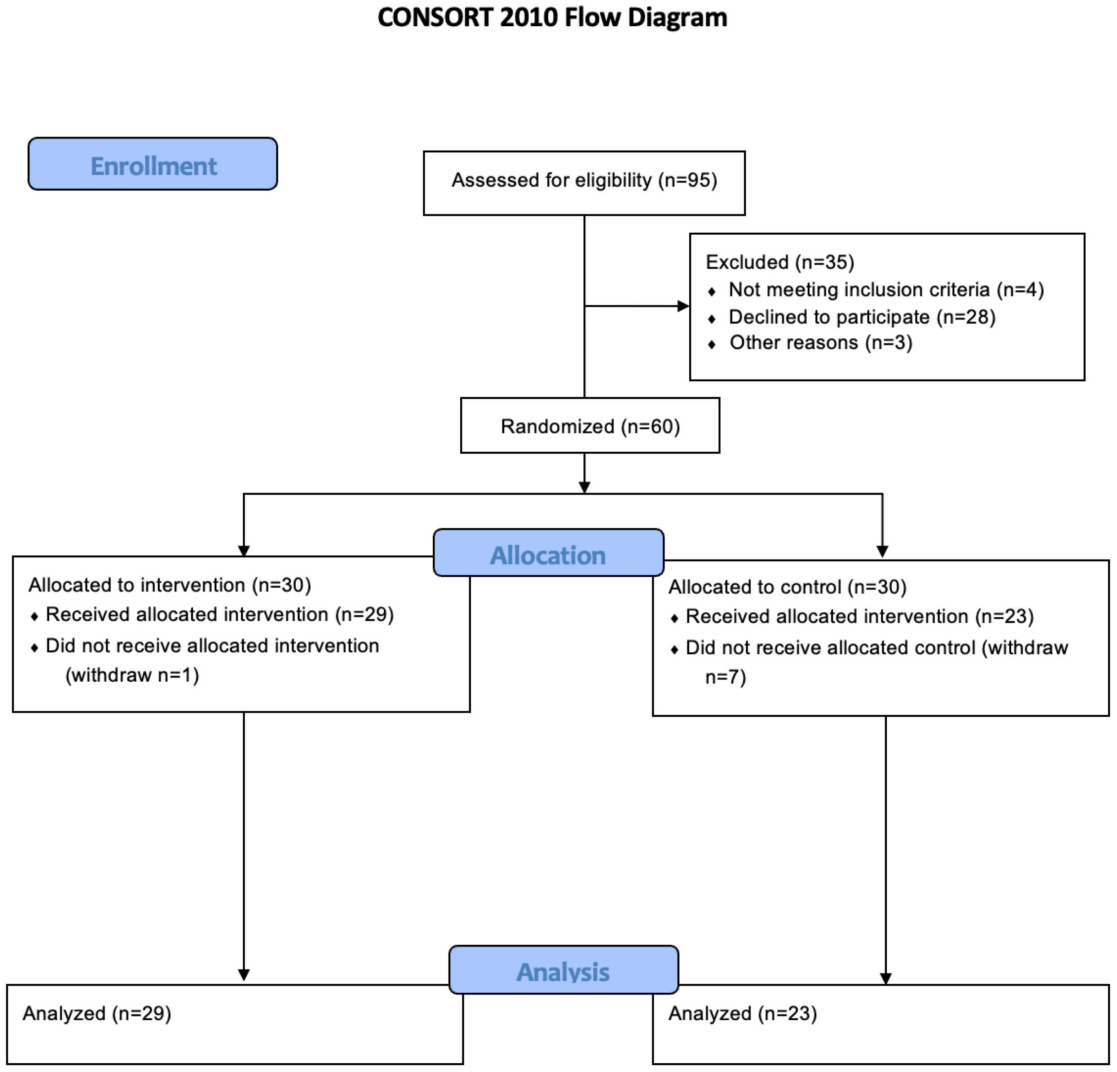
Flowchart of participant progress through the study. (Source: https://www.equator-network.org/reporting-guidelines/consort/).

An *a priori* sample size calculation using G*Power (version 3.1.9.6) indicated that 86 participants would be required to detect a medium between-group effect (Cohen’s d = 0.5) with 90% power at a two-tailed alpha level of 0.05 (12). However, due to logistical constraints and the limited number of eligible students during the study period, a total of 60 participants were recruited and randomized. Eight participants withdrew prior to analysis, resulting in a final sample of 52 (**Figure 1**). This sample allowed estimation of within-group effects and feasibility outcomes.

### Randomization

Participants were randomly assigned to the intervention or control group using a computer-generated random allocation sequence with a 1:1 allocation ratio. Due to the nature of the intervention, blinding of participants was not feasible; however, outcome assessment was conducted using self-administered standardized instruments.

### Setting

The study was conducted at the College of Applied Medical Sciences, University of Bisha, Bisha, Aseer Region, Saudi Arabia.

### Intervention

Participants in the intervention group received a single, self-selected 10-minute VR relaxation session delivered via a head-mounted display in a quiet room prior to their final practical examination. Two META Quest 2 VR headsets were used in this study. The software was provided by a local vendor and it was consisted of immersive environments designed to promote relaxation, including tropical islands and beaches, deep ocean settings, African savanna landscapes, well-known international destinations (e.g., London, New York, and the Seychelles), and outer space scenes. To minimize external distractions and enhance immersion, participants wore noise-cancelling headphones throughout the VR session. Participants were allowed to select their preferred VR environment. The control group received no VR exposure and followed their usual pre-examination routine.

### Measurements

#### Perceived stress

Perceived stress was assessed using the Perceived Stress Scale (PSS), a widely used self-report instrument designed to measure the degree to which situations in one’s life are appraised as stressful. The PSS demonstrates strong internal consistency. The scale consists of 10 items rated on a 5-point Likert scale ranging from 0 (never) to 4 (very often). Scores range from 0 to 40, with higher scores indicating greater perceived stress. Cronbach’s alpha for this scale was.87.

#### Test anxiety

Test anxiety was measured using the Test Anxiety Inventory (TAI), a validated tool assessing cognitive, emotional, and physiological aspects of anxiety in examination contexts. The TAI exhibits high internal consistency. The scale consists of 20 items rated on a 4-point Likert scale ranging from 1 (very rare) to 4 (almost always), with higher scores reflecting greater test-related anxiety. Cronbach’s alpha for this scale was.96.

#### Acceptability and feasibility of the VR intervention

Participants’ perceptions of the VR intervention were assessed in terms of both acceptability and feasibility. The Acceptability of Intervention Measure (AIM) was used to evaluate participants’ agreement with statements such as using VR to manage test anxiety is acceptable, appealing, enjoyable, and welcomed. The Feasibility of Intervention Measure (FIM) was used to assess participants’ perceptions of the VR intervention as easy to use, doable, implementable, and possible. All items were rated on a 5-point Likert scale ranging from 0 (strongly disagree) to 5 (strongly agree). Together, these instruments provided reliable and validated measures of participants’ engagement, satisfaction, and the practical implementation of the VR intervention. The Cronbach’s alpha values for AIM and FIM scales were.89 and.97, respectively.

All instruments were administered at standardized time points before and after the VR intervention (or at comparable intervals in the control group) to capture changes in stress, anxiety, acceptability, and feasibility. The selected measures are validated, reliable, and suitable for short, self-administered interventions in student populations.

### Data collection and procedure

Participants were recruited on a voluntary basis from nursing students enrolled in the Critical Care Nursing course. After confirming eligibility, written informed consent was obtained from all participants prior to study enrollment. Data collection was conducted in a quiet meeting room at the College of Applied Medical Sciences. At their scheduled time, participants completed electronic baseline (pretest) questionnaires assessing perceived stress and test anxiety using secure online survey forms before the start of the intervention or control condition. Participants allocated to the intervention group subsequently received a single, self-selected 10-minute VR relaxation session using a head-mounted display and noise-cancelling headphones, while participants in the control group followed their usual pre-examination routine. Immediately after the intervention (or equivalent waiting period for the control group), all participants completed the posttest questionnaires electronically in the same meeting room before leaving.

Outcome measures were self-administered, and no assessor blinding was applied. Acceptability and feasibility measures were administered to the intervention group following VR exposure. To ensure data integrity, pretest and posttest responses were matched using unique study identification codes. All procedures were standardized and conducted over the data collection period to ensure consistency across participants. The intervention and control conditions were scheduled to conclude at least 15 minutes before the start of the final examination to ensure that all participants had sufficient time to proceed to the examination venue and that participation in the study did not interfere with or delay their examination attendance.

### Data analysis

All statistical analyses were performed using SPSS, version 31.0.0.0 (IBM Corp., Armonk, NY, USA). Descriptive statistics were used to summarize participants’ demographic characteristics and baseline study variables. Independent t tests and chi-square tests were conducted to examine the homogeneity of participant characteristics and outcome measures between the VR intervention and control groups at baseline.

Paired t tests were used to assess within-group changes in perceived stress and test anxiety from pretest to posttest. Independent t tests were conducted to compare post-intervention differences between the intervention and control groups. Effect sizes were calculated using Cohen’s d to estimate the magnitude of intervention effects. Statistical significance was set at p <.05.

## Results

### Participant characteristics

A total of 95 eligible nursing students were assessed for eligibility. Sixty students consented and were randomized to either the VR intervention or control group. Eight participants withdrew prior to outcome assessment, resulting in a final analytic sample of 52 participants (control: n=23; intervention: n=29). No participants were lost to follow-up after allocation. Baseline characteristics showed that 55.8% of the participants were female and 44.2% were male. The majority of participants were aged 20 to 21 years (63.5%). Regarding academic year, 57.7% were in the third year, 23.1% in the second year, and 19.2% in the fourth year. The mean cumulative GPA for the sample was 3.60 ± 0.66, with 3.46 ± 0.76 for the control group and 3.71 ± 0.55 for the intervention group (**Table 1**). There were no statistically significant differences between groups in any baseline characteristic (all *p* >.05).

**Table 1:** Baseline characteristics of participants (n=52).

Characteristic	Total (n=52)	Control (n=23)	Intervention (n=29)
Sex n (%)			
Female	29 (55.8)	15 (65.2)	14 (48.3)
Male	23 (44.2)	8 (34.8)	15 (51.7)
Age (years), n (%)			
18 to <20	8 (15.4)	5 (21.7)	3 (10.3)
20 to <21	33 (63.5)	15 (65.2)	18 (62.1)
21 to <22	8 (15.4)	3 (13.0)	5 (17.2)
≥22	3 (5.8)	0 (0.0)	3 (10.3)
Academic year, n (%)			
Second year	12 (23.1)	5 (21.7)	7 (24.1)
Third year	30 (57.7)	13 (56.5)	17 (58.6)
Fourth year	10 (19.2)	5 (21.7)	5 (17.2)
Cumulative GPA, mean ± SD	3.60 ± 0.66	3.46 ± 0.76	3.71 ± 0.55

Note: n, number of participants; %, percentage; No significant differences between groups (all p >.05).

### Perceived stress

Within-group analyses demonstrated no significant change in perceived stress scores in the control group from pretest to posttest (*p* = .762, Cohen’s *d* = 0.06). In contrast, the intervention group showed a statistically significant reduction in perceived stress following the VR relaxation session, with scores decreasing from pretest to posttest (*p* = .014, Cohen’s *d* = 0.49). Between-group comparisons at posttest did not reach statistical significance (*p* = .916), likely reflecting variability in posttest scores and the modest sample size due to the exploratory nature of this study (**Table 2**).

**Table 2:** Within-group changes in perceived stress and test anxiety.

Outcome	Group	Pretest M ± SD	Posttest M ± SD	*p* value	Cohen’s d
PSS	Control	27.39 ± 7.72	26.52 ± 10.29	.762	0.06
	Intervention	29.83 ± 3.36	26.76 ± 5.51	.014	0.49
TAI	Control	62.91 ± 14.41	55.70 ± 22.01	.179	0.29
	Intervention	65.76 ± 10.61	53.24 ± 14.35	.002	0.64

M, Mean; SD, Standard Deviation.

### Test anxiety

In the control group, test anxiety scores decreased from pretest to posttest; however, this change was not statistically significant (*p* = .179, Cohen’s *d* = 0.29). Conversely, participants in the VR intervention group demonstrated a significant reduction in test anxiety, with scores declining from pretest to posttest (*p* = .002, Cohen’s *d* = 0.64), see **Table 2**. No statistically significant between-group differences were observed at posttest (p = .630).

### Acceptability and feasibility of the VR intervention

Participants in the intervention group reported favorable perceptions of the VR relaxation experience. The AIM yielded a mean score of 13.03 ± 3.07, indicating positive emotional engagement and satisfaction with the intervention. The FIM demonstrated a high mean score of 15.69 ± 1.07, suggesting that the VR intervention was easy to use, feasible to complete, and appropriate for implementation immediately prior to examinations, see **Table 3**.

**Table 3:** Acceptability and feasibility of the VR intervention.

Measure	M ± SD
AIM	13.03 ± 3.07
FIM	15.69 ± 1.07

M, Mean; SD, Standard Deviation; AIM, Acceptability of Intervention Measure; FIM, Feasibility of Intervention Measure.

## Discussion

This RCT demonstrates that a brief, 10-minute self-selected immersive VR relaxation session is a feasible and highly effective intervention for reducing acute academic stress and test anxiety in nursing students. The primary finding is the robust reduction in perceived stress among the intervention group, evidenced by a moderate effect size (Cohen’s *d* = 0.49), alongside a moderate to large reduction in test anxiety (Cohen’s *d* = 0.64). Notably, the control group exhibited a non-significant increase in stress and anxiety markers. These results align with the growing body of literature supporting VR as an effective tool in healthcare and nursing education (6, 13, 14).

Participants in the intervention group demonstrated a significant reduction in perceived stress and test anxiety from pretest to posttest, while the control group showed no significant changes. These results suggest that VR relaxation may be an effective, brief intervention to reduce acute academic stress among nursing students. The results are consistent with prior studies demonstrating the effectiveness of VR-based relaxation interventions for stress and anxiety management in academic and clinical populations (5, 6).

Although no physiological measures were used in this study, the observed efficacy of the intervention could be explained by several psychological and physiological mechanisms. First, the high levels of acceptability (AIM) and feasibility (FIM) suggest that the immersive nature of VR facilitates a rapid transition from a high-arousal state to a state of “presence” and relaxation. By engaging multiple sensory modalities (i.e., visual and auditory) the VR environment likely interrupts the cycle of cognitive rumination often associated with pre-exam anxiety (15). Furthermore, the element of user agency played a critical role.

Consistent with the Theory of Self-Determination, allowing participants to self-select their environment (e.g., beach, forest, or space) likely enhanced their sense of autonomy and control, which are factors often diminished in high-stakes testing environments (16). This aligns with findings suggesting that user choice correlates with stronger emotional regulation and a deeper sense of presence (11, 17).

The immersive nature of VR, especially when combined with nature sounds and ambient visuals, appears to stimulate relaxation through sensory distraction and emotional engagement (10). The combination of nature-based stimuli and ambient soundscapes serves as a “digital restorative environment,” theoretically activating parasympathetic responses similar to those observed in traditional mindfulness or nature-exposure therapies, but within a timeframe feasible for a busy academic schedule (18, 19).

The present study demonstrated high feasibility and efficacy of VR use in a Saudi educational setting, where psychological stress management interventions remain underutilized. The findings have important implications for nursing education and student well-being. Integrating brief, technology-based relaxation interventions into academic programs could help mitigate stress and test anxiety commonly experienced by nursing students, especially during high-stakes practical assessments. These interventions may be implemented in simulation centers, clinical skills laboratories, or student study environments to help students manage stress and anxiety. This could be achieved by integrating VR sessions into pre-clinical exam preparation or providing access to stress-reduction VR content in student wellness programs. The portability and accessibility of standalone VR headsets make this approach scalable, cost-effective, and adaptable to various educational settings.

Future research should extend these findings by exploring the potential application of VR-based stress reduction among practicing nurses in clinical environments. In addition, further studies are needed to examine possible dose–response relationships, including the optimal duration and frequency of VR exposure, as well as whether the timing of the intervention relative to examinations (e.g., immediately before vs earlier in the day) influences its effectiveness.

## Limitations

The sample size was relatively small, and all outcomes were self-reported, which may introduce response bias. A lack of between-group comparison of change scores limits the ability to make strong causal inferences, and the short duration of the intervention precludes assessment of sustained effects. Future research should replicate these findings with larger, more diverse samples, include reliable physiological measures such as heart rate variability, and explore long-term retention of relaxation benefits. Although participants were allowed to select their preferred VR environment, scene selection data were not systematically collected. Future studies are encouraged to examine whether different types of VR environments (e.g., nature-based versus urban or tourism-based settings) differentially influence stress reduction outcomes. Despite these limitations, the current results provide promising preliminary evidence supporting the use of short, immersive VR relaxation experiences as an effective and engaging tool for managing acute stress and test anxiety among nursing students.

## Conclusion

This study provides preliminary evidence that a brief, self-selected immersive VR relaxation session represents a promising, feasible approach for mitigating academic stress and test anxiety among nursing students. As nursing education continues to operate within high-pressure learning environments, the integration of digital therapeutics such as VR may offer a scalable strategy to support student well-being and cognitive readiness. By fostering emotional regulation through innovative technologies, such interventions have the potential to enhance both individual student resilience and preparedness within the future nursing workforce.

## Data Availability

The raw data supporting the conclusions of this article will be made available by the authors, without undue reservation.
